# Multidisciplinary analysis of the prognosis and biological function of NUBPL in gastric cancer

**DOI:** 10.3389/fimmu.2025.1603898

**Published:** 2025-06-05

**Authors:** Luqian Liu, Fengyu Zhang, Jiaye Yu, Pingping Dong, Jing Deng, Weiwei Liu, Xinlei Cao, Juanjuan Huang, Xiaoming Lin, Xiangyang Xue

**Affiliations:** ^1^ Wenzhou Collaborative Innovation Center of Gastrointestinal Cancer in Basic Research and Precision Medicine, Wenzhou Key Laboratory of Cancer-related Pathogens and Immunity, Department of Microbiology and Immunology, Institute of Molecular Virology and Immunology, School of Basic Medical Sciences, Wenzhou Medical University, Wenzhou, China; ^2^ School of Biomedical Engineering, Tianjin Medical University, Tianjin, China; ^3^ Department of Thoracic Surgery, The First Affiliated Hospital of Wenzhou Medical University, Wenzhou, Zhejiang, China; ^4^ Department of Biomedical Sciences, Faculty of Health Sciences, University of Macau, Taipa, Macao SAR, China

**Keywords:** gastric cancer, NUBPL, disulfidptosis, biomarker, immunotherapy, machine learning

## Abstract

**Background:**

Researchers are currently concentrating on molecular markers and potential therapeutic targets associated with gastric cancer in light of recent developments in precision medicine and molecular biology. Disulfidptosis was first proposed in 2023 as a novel programmed cell death mode associated with the cytoskeleton. Disulfidptosis-related proteins are essential for the preservation of protein stability and abnormal expression of disulfidptosis-related genes may be linked to cancer development and drug resistance.

**Materials and method:**

The gastric cancer transcriptomic data were retrieved from TCGA database, and disulfidptosis-related genes were identified through literature search. Utilizing machine learning methods such as LASSO, Random Forest (RF), Boruta, SVM-RFE, and XGBoost, the disulfidptosis-related gene NUBPL was determined as a potential predictor for gastric cancer. PPI network was constructed, and the GO database as well as the KEGG database were employed to analyze the protein interactions and pathway enrichment of NUBPL in gastric cancer. Meanwhile, the ESTIMATE algorithm was used for immune infiltration analysis and prediction of immunotherapy response, and the Genomics of Drug Sensitivity in Cancer (GDSC) database was utilized for the drug sensitivity analysis of NUBPL. The role of NUBPL in gastric cancer and its inhibition of disulfidptosis were validated using molecular biological methods.

**Results:**

The aberrant expression of NUBPL significantly impacts the prognosis of gastric cancer and modulates metabolic and immune-related pathways. In patients with elevated NUBPL expression levels, a reduced number of CD8-positive T cells is associated with adverse prognosis and gastric cancer progression. Elevated NUBPL expression levels can impair the function of chemokines. Moreover, patients with lower NUBPL expression levels exhibit better responses to immunotherapy. We have also identified drugs such as QS11, Imatinib, and AS601245 as potential inhibitors of NUBPL. *In vitro* experiments have shown that NUBPL affects the invasion and migration of gastric cancer cells, rather than proliferation and apoptosis, by regulating the PPP pathway and inhibiting disulfidptosis.

**Conclusion:**

This study underscores the pivotal role of NUBPL in gastric cancer progression and highlights its significance as a potential target for targeted therapy and immunotherapy gastric cancer, NUBPL, disulfidptosis, biomarker, immunotherapy, machine learning

## Introduction

Disulfidptosis, a distinctive type of cellular demise, has attracted considerable interest in the management of gastric cancer (1). It was first proposed in 2023 by Professor Gan’s team at the MD Anderson Cancer Center in the United States. It is an emerging type of programmed cell death associated with glucose deprivation and is distinct from apoptosis, necrosis, autophagy, and ferroptosis (2). This process entails swift cellular demise triggered by disulfide stress during glucose deprivation, intimately associated with many proteins connected to stomach cancer. These proteins may be crucial to the etiology of gastric cancer, especially in tumor proliferation and immunological responses (3). Notwithstanding progress in treating locally advanced gastric cancer, including the integration of surgery and adjuvant hyperthermic intraperitoneal chemotherapy, the prognosis for patients with gastric cancer continues to be unfavorable, with numerous individuals succumbing to disease progression within two years following surgery (4, 5). Moreover, patients with stomach cancer frequently encounter several comorbidities, including peritoneal infection, pulmonary embolism, and tumor hemorrhage, all of which exacerbate mortality risk. Consequently, comprehensive investigation into the function of disulfidptosis-regulating proteins in gastric cancer may uncover prospective diagnostic and therapeutic targets, thereby enhancing patient survival and quality of life.

The disulfidptosis mechanism affects gastric cancer cell mortality through various pathways. The phenomenon is chiefly attributed to the overexpression of solute carrier family 7 member 11 (SLC7A11) and the cellular environment during glucose deprivation. During glucose deprivation, cancer cells exhibiting heightened SLC7A11 expression excessively uptake cystine, leading to NADPH depletion that impedes the conversion of cystine to cysteine (2, 6). Disulfidptosis specifically modifies the dynamics of actin cytoskeletal proteins, leading to the disintegration of the actin network and, ultimately, cellular demise. Moreover, research indicates that the inhibition of the WAVE regulatory complex can mitigate cell death induced by disulfidptosis, whereas sustained Rac activation exacerbates this phenomenon (7, 8). NUBPL is a protein that helps put together the main respiratory chain component of mitochondria, human mitochondrial complex I. Compared to normal tissue, colorectal cancer (CRC) exhibits much higher NUBPL expression, which is highly correlated with lymph node metastases and advanced stages (9, 10). NUBPL promote EMT and tumor metastasis. However, our understanding of NUBPL’s exact function in gastric cancer is still limited (9, 11). The pentose phosphate pathway (PPP) plays a crucial role in maintaining the cellular antioxidant defense by providing NADPH, which is essential for redox homeostasis and biosynthesis (12). NADPH also helps recycle oxidized glutathione (GSSG) into reduced glutathione (GSH), thereby scavenging reactive oxygen species (ROS) and protecting cells from oxidative damage (13). The PPP is over-activated in tumor cells. Some studies have shown that the PPP promotes the proliferation and development of tumors by providing NADPH and intermediate metabolites (14, 15). However, there has been relatively little research on whether NUBPL affects the PPP.

This study employed machine learning to discover genes associated with disulfidptosis in gastric cancer. Abnormal NUBPL expression may facilitate gastric cancer progression and hence influence patient prognosis. We found evidence that NUBPL could be a good target for treating stomach cancer. We also determined how NUBPL regulates disulfidptosis in gastric cancer and evaluated its effects on malignant phenotype in gastric cancer.

## Materials and methods

### Data sources

The gastric cancer transcriptomic data set (STAD) was obtained from the TCGA website. The following were the selection criteria: (1) STAD patient primary tumor samples together with paired normal tissue samples; (2) sequencing samples obtained from patient frozen tissues; (3) survival data that is readily available. There were a grand total of 383 tumor samples along with 36 nearby normal samples. A total of 29 genes associated with disulfide bonds were found through a literature review.

### Machine learning for biomarker selection

Two sets of samples were randomly divided into training and testing groups using a 5:5 ratio. A LASSO regression analysis was performed on the disulfide-related genes using the “glmnet” package. The LASSO method effectively screens variables and reduces model complexity through parameter regularization, thereby mitigating overfitting. The degree of regularization is controlled by the tuning parameter λ, which penalizes coefficient magnitudes and ultimately yields a sparse model with a reduced set of predictive variables. To optimize model performance, we implemented k-fold cross-validation (k = 10) using R software(version 4.4.1). The optimal λ value was selected based on the minimum mean cross-validated error, ensuring robust predictor selection while maintaining model generalizability. A ROC curve was plotted using the “survivalROC” package to evaluate the model’s predictive accuracy. A higher AUC indicates a more accurate model. For the Random Forest model, we implemented the “rfsrc” function from the randomForestSRC package in R, with two critical parameters: ntree (number of trees) set to 1000 and nodesize (minimum terminal node size) set to 50. The selection of these parameters was designed to optimize model performance. To rigorously evaluate feature importance, we conducted an extensive 1000-iteration Monte Carlo simulation using the same package, which enabled robust ranking of prognostic genes based on their variable importance measures. Thanks to this, we were able to select features. The last thing we did was search for genes with a relative importance higher than zero. We used a Venn diagram to show how the two sets of critical genes overlapped after we intersected them. At the same time, this study comprehensively applied the Boruta algorithm, the Support Vector Machine Recursive Feature Elimination (SVM-RFE) algorithm, and the eXtreme Gradient Boosting (XGBoost) algorithm for feature selection and importance assessment.The Boruta algorithm screens out important features step by step by comparing the importance of the original features with that of the shadow features. The SVM-RFE algorithm ranks the feature importance of gene expression data through 10-fold cross-validation. The XGBoost algorithm trains the model and evaluates the feature importance by setting parameters (such as the maximum depth of the tree being 6, the learning rate being 0.5, and the number of iterations being 25). In addition, the caret package and the DALEX package are used to train, interpret, and evaluate the performance of multiple models. Receiver Operating Characteristic (ROC) curves and residual plots are plotted, and the top 10 genes in terms of importance for each model are outputted.

### Association analysis of key gene expression with clinical factors

By investigating the relationship between crucial gene expression and prognosis and clinical variables, we sought to clarify the clinical predictive relevance of these genes and offer new perspectives for the management of at-risk individuals. The Wilcoxon test was utilized to investigate significant differences in gene expression between gastric cancer tissues and their adjacent normal counterparts. Utilizing an optimal cutoff point, gene expression levels were classified into high and low categories with the help of the surv_cutpoint function from the survminer package. To assess the correlation between gene expression and patient survival, Kaplan-Meier survival curves were constructed. The association between the expression of two key genes and the staging of gastric cancer was examined using the GEPIA website (http://gepia.cancer-pku.cn/). Furthermore, boxplots were created using the ggpubr package in R, and the Wilcoxon test was utilized to evaluate the statistical significance of the association between essential gene expression and prognosis in susceptible populations.

### Functional enrichment analysis

To identify key biological pathways associated with NUBPL expression, we performed comprehensive functional enrichment analyses. Differential gene expression analysis between NUBPL high and low expression groups was conducted using the DESeq2 algorithm, with significant differentially expressed genes (DEGs) defined as those meeting the threshold of |log2 fold change| > 1 and adjusted *p*-value < 0.05. The results were visualized using volcano plots.

For functional characterization, we employed the “clusterProfiler” R package to perform Gene Ontology (GO) and Kyoto Encyclopedia of Genes and Genomes (KEGG) pathway enrichment analyses on the identified DEGs. Significant terms were selected using a q-value cutoff of 0.05 with Benjamini-Hochberg (BH) correction for multiple testing.

To explore protein-protein interaction networks, we selected the top 100 most significant DEGs (ranked by q-value) and constructed protein-protein interaction (PPI) networks using the ClueGO plugin in Cytoscape software (version 3.9.1), enabling visualization of key gene functions within biological networks.

Gene Set Enrichment Analysis (GSEA) was subsequently performed using the “clusterProfiler” package to identify pathways exhibiting activated states in the NUBPL high-expression group.

For comprehensive pathway assessment, we applied Gene Set Variation Analysis (GSVA) to calculate enrichment scores for both GO terms and KEGG pathways. The correlation between these pathway scores (including metabolic and immune-related pathways) and NUBPL expression levels was evaluated using Spearman’s rank correlation analysis.

### Immune infiltration analysis

To investigate the correlation between key gene expression and immune cell infiltration in the tumor microenvironment, we employed the ESTIMATE algorithm to analyze stromal and immune cell infiltration levels in gastric cancer tissue. The ESTIMATE algorithm calculates three distinct scores - Stromal Score, Immune Score, and ESTIMATE Score - serving as robust indicators for predicting tumor purity and evaluating stromal/immune cell infiltration levels in tumor tissues. By computing these stromal and immune scores using the R package “ESTIMATE”, we systematically analyzed whether NUBPL differential expression correlates with immune infiltration.

For comprehensive immune profiling, we utilized the CIBERSORT algorithm (https://cibersortx.stanford.edu/) to quantify the proportions of 22 tumor-infiltrating immune cell subtypes in each sample. This advanced machine learning approach employs a deconvolution method to infer immune cell composition from complex tumor tissue gene expression profiles. Differential immune cell abundance between NUBPL expression groups was visualized using violin plots generated with the R package “ggplot2”, with statistical significance defined as *p*<0.05.

Regarding immunotherapy relevance, immune checkpoint blockade therapy operates through T-cell activation mechanisms. Specifically, programmed death receptor/ligand blockade enhances host immune system’s anti-tumor activity by inhibiting their interaction. We examined expression patterns of four critical immune checkpoint molecules across NUBPL expression groups.

Furthermore, we conducted Spearman correlation analysis using the TISIDB database (https://cis.hku.hk/TISIDB/) to explore potential associations between NUBPL expression and chemokine profiles in gastric cancer, providing additional insights into its immunomodulatory role.

### Prediction of immune therapy response

The relationship between key gene expression levels and immune therapy response was comprehensively analyzed in this study using three methods: IPS score, TIDE score, and Submap. The TCIA database (https://tcia.at/) was used to download IPS scores, which evaluate responses to CTLA-4 and PD-1 blockade therapies. A worse response to immune therapy is suggested by a higher TIDE score. In addition, we implemented the Submap algorithm (http://cloud.genepattern.org/gp) to anticipate the potential responses of risk groups to PD-1 and anti-cytotoxic T lymphocyte-associated antigen 4 (CTLA-4) therapies. In addition, the evaluation of the immune response in this study is derived from cell lines.

### Drug sensitivity analysis

Drug genomics data from the Cancer Drug Sensitivity Genomics (GDSC) database was employed to predict the relationship between essential gene expression levels and drug sensitivity. In this investigation, drug molecule SDF files were downloaded from the PubChem database (https://pubchem.ncbi.nlm.nih.gov/) and incorporated into AutoDock Vina software (version 1.5.7) 34,35. Subsequently, atom types were ascribed and atomic charges were incorporated. The results were stored in PDBQT format. The target protein files for docking, which were obtained from the UniProt database (https://www.uniprot.org/), were purged of water molecules and extraneous small molecules. Subsequently, the protein files were imported into AutoDock Vina, hydrogen atoms were incorporated, and the files were saved in PDBQT format. Ultimately, the binding free energy was determined by aligning the drug molecules with the target proteins using AutoDock Vina. A reduced binding energy is indicative of a stronger affinity between the drug molecule and the target protein. Following the selection of potential drugs based on binding energy and binding site analysis, the three-dimensional (3D) and two-dimensional (2D) structures of the drug-protein binding pocket were visualized using Discovery Studio software 36.

### Cell culture

Two GC cell lines, AGS and MKN-45, were purchased from the Cell Bank of the Chinese Academy of Sciences (Shanghai, China). These cell lines were grown in RPMI-1640 (Gibco, Thermo Fisher Scientific) medium supplemented with 10% fetal bovine serum (FBS, Gibco) at 37°C.

### Construction and verification of NUBPL overexpression cell line

The full-length NUBPL cDNA was cloned into a suitable vector (pcDNA3.1) to construct NUBPL overexpression plasmids. Then, using the transfection reagent Lipofectamine 2000, the plasmid was transferred into the correct cells (AGS and MKN-45). Using Western blotting (WB) to detect protein levels, respectively, we confirmed overexpression of NUBPL.

### Cell invasion and migration assays

To assess the cells’ capacity for invasion and migration, a Transwell chamber with or without a Matrigel coating was used. The cells were seeded in 6-well plates overnight and transfected with siRNA or NUBPL plasmid respectively. After 24 hours, the cells were digested with trypsin. In the upper chamber, 1×10^5^ transfected cells were suspended in 200 μl of serum-free medium, and 600 μl of complete medium containing 10% fetal bovine serum (FBS) was added to the lower chamber. Post a 24 h incubation period, the permeated cells were immobilized, dyed with crystal violet, and enumerated using a microscope.

### Western blotting

Protease and phosphatase inhibitors were added to RIPA buffer to prepare cell lysates. A BCA protein assay reagent was employed to quantify protein concentrations. The protein sample (30 µg) underwent SDS-PAGE separation, was then transferred to PVDF membranes, and was probed with primary antibodies recognizing proteins linked to sulfur-mediated cellular demise. This was followed by the addition of secondary antibodies. An ECL detection system was employed to identify protein bands.

### Data analysis

GraphPad Prism 9.0 (GraphPad, San Diego, CA) and FlowJo (BD, USA) software were used for data analysis. The findings are shown as mean ± SD, and each experiment was performed three times. The statistical analysis for comparing different groups relied on the t-test method; significance was deemed to be achieved when the p-value dipped below 0.05. Each experiment was meticulously repeated at least three times to ensure that the results were consistent and reproducible.

## Results

### Identification of hub genes using machine learning

**Figure 1:**
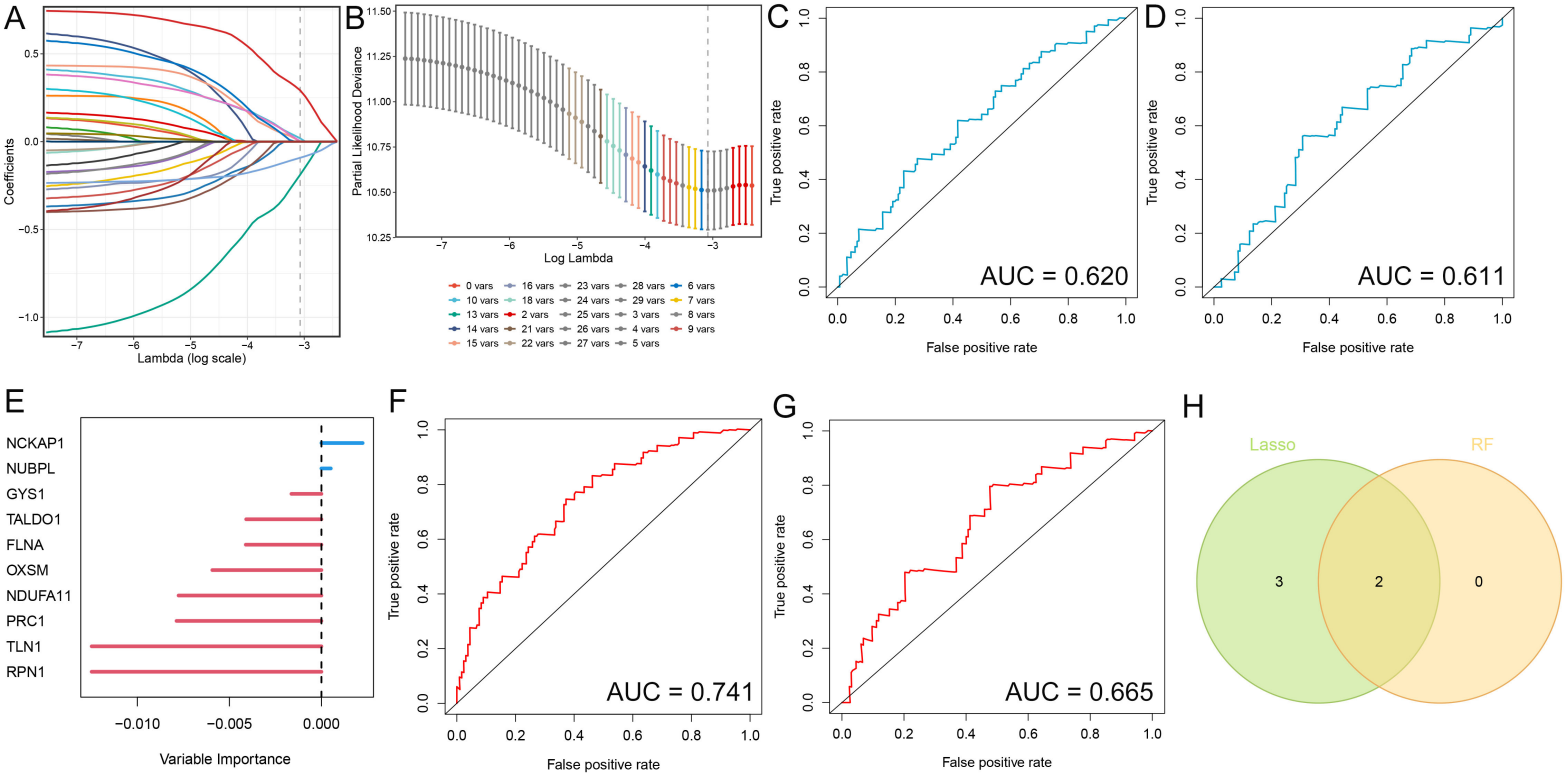
Identification of diagnostic genes employing two machine learning algorithms. **(A)** Variable selection utilizing Lasso regression, illustrating the variations in variable coefficients. **(B)** Selection procedure for the optimal parameter λ in the Lasso regression model via cross-validation. **(C, D)** ROC curves for the test and validation sets in the Lasso model. **(E)** Analysis of gastric cancer risk utilizing Random Survival Forest methodology. **(F, G)** ROC curves for the test and validation sets in the Random Forest model. **(H)** Venn diagram illustrating candidate diagnostic genes identified by both algorithms.

The identification of hub genes that are associated with gastric cancer through the use of LASSO and random forest machine learning methods. In LASSO regression, λ is the tuning parameter that controls the strength of regularization (L1 penalty). The dotted vertical lines are drawn at optimal values by using the minimum criteria. An optimal λ value was selected for the LASSO model by using 10-fold cross-validation via minimum criteria. The result showed a total of 5 significant gene signatures were eventually determined to establish the model on the basis of log (λ)= -3.072645(**Figures 1A, B**). The diagnostic value of these genes was further evaluated by plotting ROC curves for the training and validation sets, which yielded AUC values of 0.620 and 0.611, respectively (**Figures 1C, D**). We identified two critical genes (**Figure 1E**) through the Random Forest method. The Area Under the Curve (AUC) for the training and validation datasets were 0.741 and 0.665, respectively (**Figures 1F, G**). We discovered that these genes exhibited a relatively high predictive accuracy for the disease (AUC > 0.6). NCKAP1 and NUBPL were the intersection genes identified by both methods, as illustrated by a Venn diagram.

**Figure 2:**
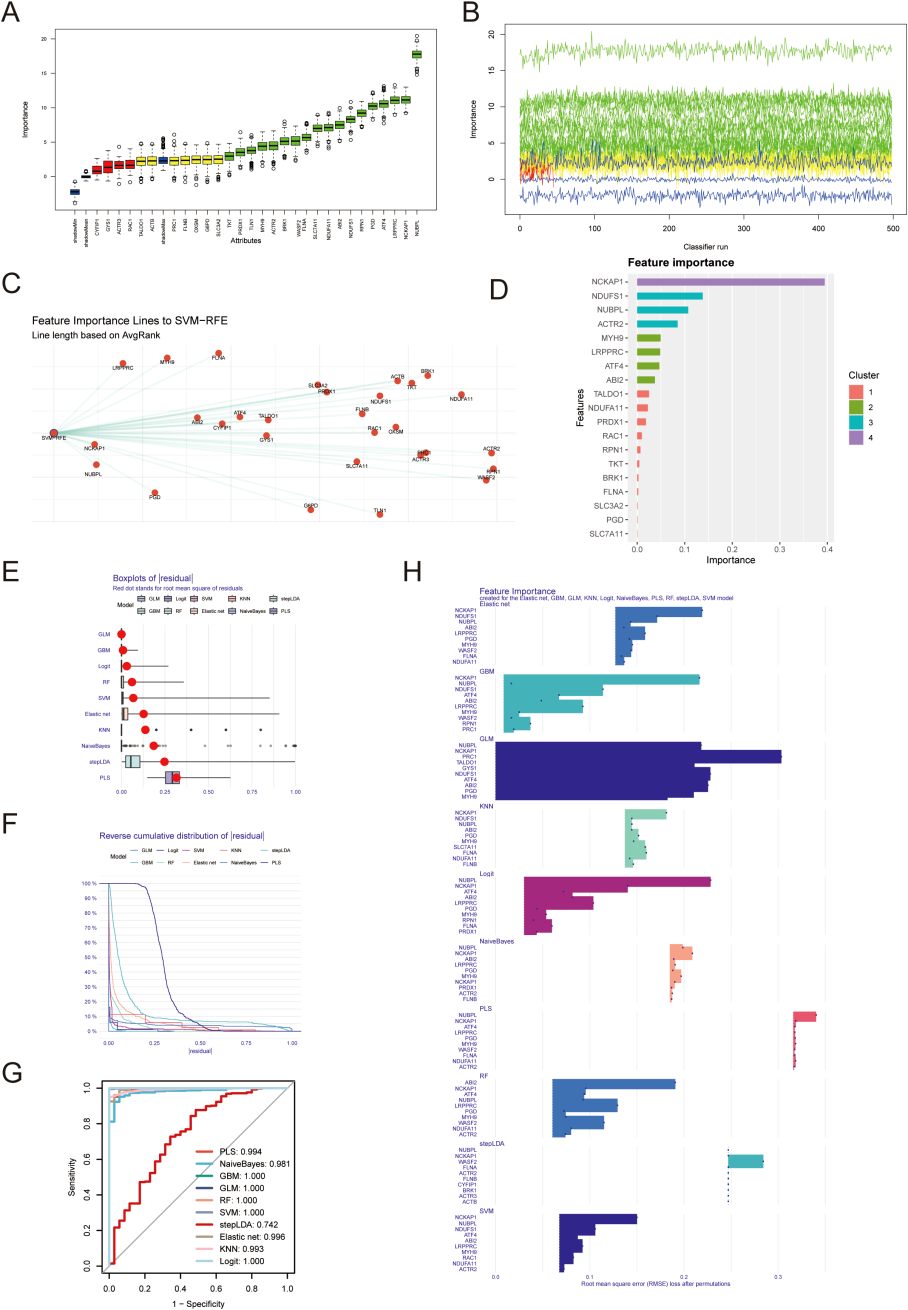
Identification of diagnostic genes employing machine learning algorithms. **(A, B)** 23 important characteristic genes were identified by Boruta algorithm. **(C)** Support vector machine recursive feature elimination (SVM-RFE) algorithm was used to identify the top 10 genes in gene expression data. **(D)** xgboost algorithm was used to obtain 19 important genes. **(E)** The residual box plot shows the median, quartile range, outlier and other information of the residual. **(F, G)** The inverse cumulative distribution of sample residuals and ROC curve shows that almost 100% of sample residuals are concentrated near 0. **(H)** Each model outputs the top10 most important genes.

Through the Boruta algorithm, we identified 23 relatively significant features (PRC1, FLNB, OXSM, G6PD, SLC3A2, TKT, PRDX1, TLN1, MYH9, ACTR2, BRK1, WASF2, FLNA, SLC7A11, ABI2, NDUDS1, RPN1, PGD, ATF4, LRPPRC, NCKAP1, NUBPL) (**Figures 2A, B**). Differential analysis was performed on these 23genes, revealing differences between the normal and tumor groups. If the values in the normHits column are very close to 1, the p-values or significance from the differential analysis tend to be highly favorable. Conversely, if the values are far from 1, the significance is less pronounced. We also employed the Support Vector Machine-Recursive Feature Elimination (SVM-RFE) algorithm to identify and rank the most significant features in the gene expression data. The top 10 feature genes based on their importance are as follows: NCKAP1, NUBPL, LRPPRC, PGD, MYH9, ABI2, FLNA, CYFIP, ATF4, GYS1 (**Figure 2C**). Utilizing the XGBoost algorithm, we identified 19 important features, including: NCKAP1, NDUFS1, NUBPL, ACTR2, MYH9, LRPPRC, ATF4, ABI2, TALDO1, NDUFA11, PRDX1, RAC1, RPN1, TKT, BRK1, FLNA, SLC3A2, PGD, SLC7A11. Among them, NCKAP1, NDUFS1 and NUBPL was the three genes with the highest importance score (**Figure 2D**). Consequently, integrating the results from these machine learning approaches, we have selected NCKAP1 and NUBPL as the key genes for further investigation.

The residual box plot illustrates the median, interquartile range (IQR, the difference between the third and first quartiles), and outliers of the residuals (**Figure 2E**). When the reverse cumulative distribution plot of sample residuals shows that almost 100% of the residuals cluster around zero, this typically indicates that the model’s predictions are very close to the actual observed values, allowing for highly accurate predictions for most data points the prediction error is minimal (**Figure 2F**). The ROC curve yields consistent results, suggesting excellent predictive performance of the model (**Figure 2G**). Each model outputs the top 10 most significant genes, and we observe that multiple algorithms consistently identify NUBPL and NCKAP1 as having notable relative importance (**Figure 2H**).

### Association between hub genes and clinical pathological features in STAD patients

**Figure 3:**
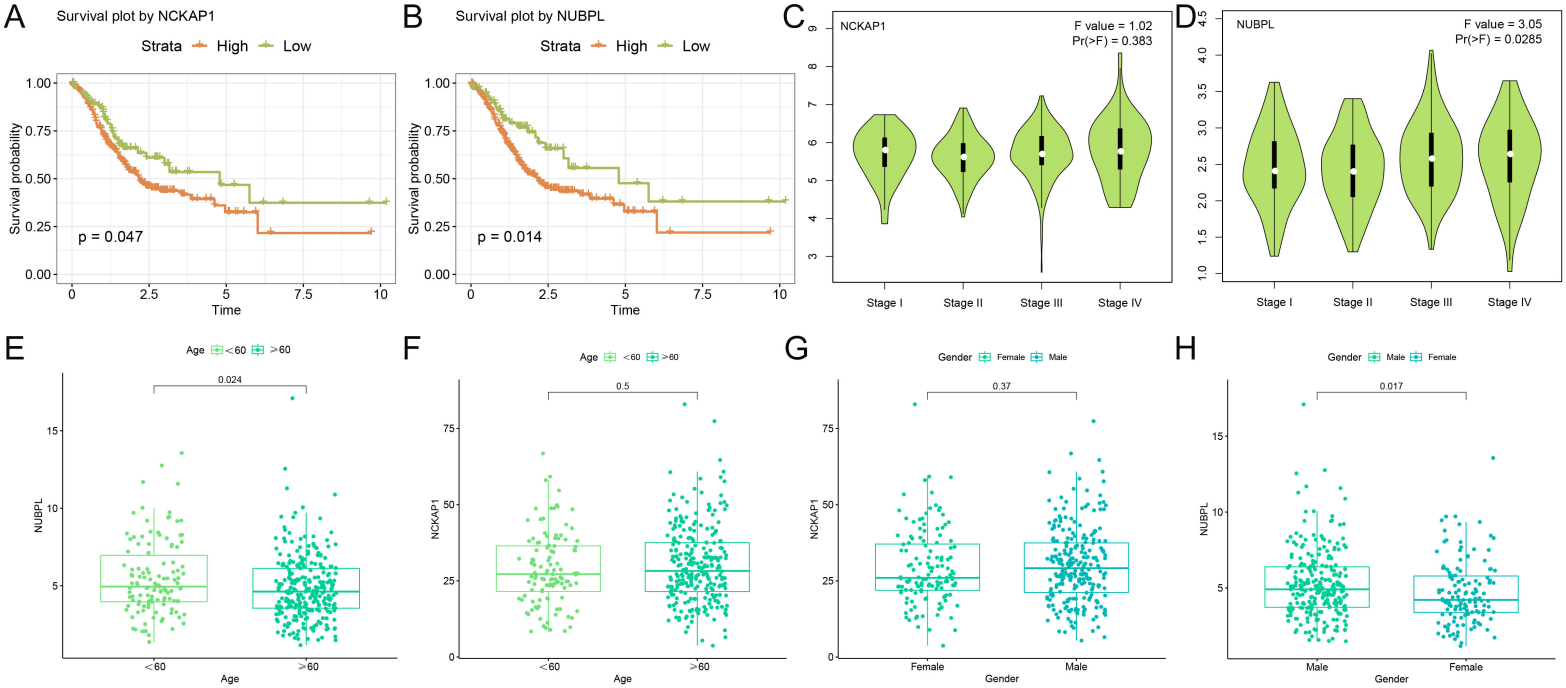
Clinical examination of hub genes **(A, B)** Kaplan-Meier survival curves illustrating the survival rates of high and low expression cohorts for NCKAP1 and NUBPL. **(C, D)** Expression levels of NCKAP1 and NUBPL at various stages of gastric cancer. **(E, F)** Variations in the expression of NCKAP1 and NUBPL among gastric cancer patients of varying ages. **(G-H)** Association between NCKAP1 and NUBPL Expression Levels and Gender.

By analyzing the average level of NCKAP1 expression, we divided the STAD patients into two groups: those with high and those with low expression. We then performed a Kaplan-Meier survival study to determine if there’s a notable link between NCKAP1 and the overall survival rate in STAD patients. We discovered that a poor prognosis was associated with high expression of NCKAP1 (*p*=0.047, **Figure 3A**). Additionally, the survival curve for NUBPL demonstrated that patients with elevated NUBPL expression had a significantly shorter overall survival (OS) (*p*=0.014, **Figure 3B**). In STAD tissues, both NCKAP1 and NUBPL showed significantly higher expression levels compared to adjacent normal tissues(*p*<0.05, **Supplementary Figure S1**). NCKAP1 expression did not exhibit any significant variation across STAD stages (*p*=0.383, **Figure 3C**); however, NUBPL expression was significantly elevated in stages III and IV (*p*=0.0285, **Figure 3D**). Furthermore, box plots demonstrated that patients under the age of 60 exhibited a higher level of NUBPL expression (*p*=0.024, **Figure 3E**), and male STAD patients exhibited a significantly higher level of NUBPL expression than female patients (*p*=0.017, **Figure 3H**). There was no significant correlation between NCKAP1 expression and gender or age factors (**Figures 3F, G**).

### Functional enrichment and pathway regulation analysis of NUBPL

**Figure 4:**
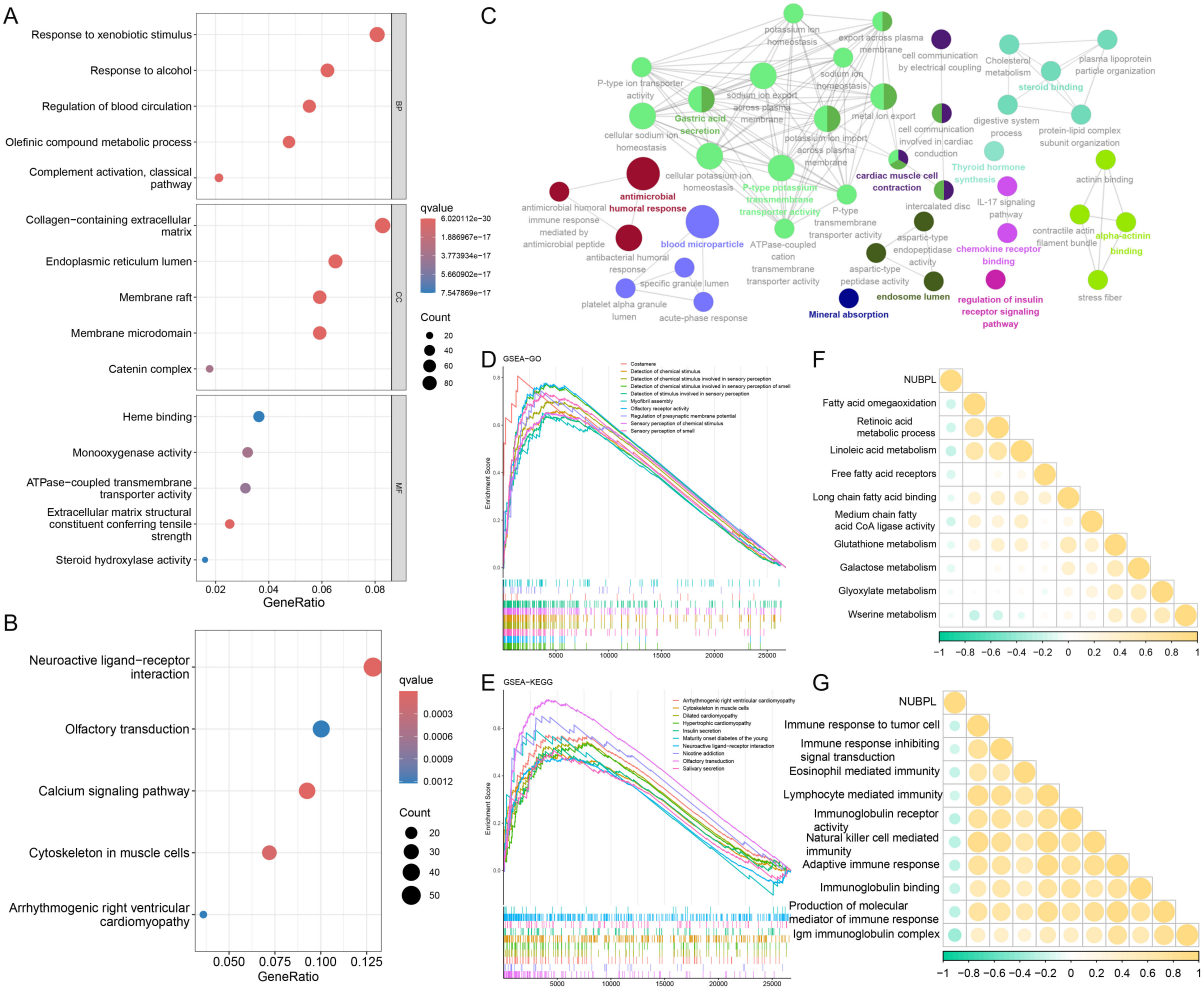
Biological functions of NUBPL. **(A, B)** GO and KEGG enrichment analysis of differentially expressed genes in high versus low expression cohorts of NUBPL. **(C)** ClueGO analysis of the top 100 differentially expressed genes (DEGs) **(D, E)** GSEA analysis of Gene Ontology and KEGG gene sets. **(F, G)** Quantification of pathway correlation using the GSVA algorithm.

Utilizing the NUBPL median expression as a criterion, we segregated the samples into high-expression and low-expression subsets before proceeding with differential investigations. The criteria for differential genes were |logFC| > 1 and adj. *p* < 0.05, resulting in 2,345 upregulated and 281 downregulated genes (**Supplementary Figure S2**). The functional differences in genes between the NUBPL high and low expression cohorts were determined using functional enrichment analysis. The GO enrichment bubble plot demonstrates that these differential genes are primarily enriched in pathways related to the modulation of blood circulation, the response to xenobiotic stimuli, and the response to alcohol (**Figure 4A**). The differentially expressed genes are linked to pathways such as neuroactive ligand-receptor interaction and calcium signaling, as demonstrated by the KEGG enrichment analysis (**Figure 4B**). Our results also indicates that aberrant expression of NUBPL primarily influences pathways associated with P-type potassium transmembrane transporter activity, gastric acid secretion, and steroid binding (**Figure 4C**). The GSEA results indicate that pathways including Detection of Chemical Stimulus in Sensory Perception, Cytoskeleton in Muscle Cells, and Neuroactive Ligand-Receptor Interaction are activated in the high expression group of NUBPL (**Figures 4D, E**). The correlation heatmap indicates that NUBPL expression is correlated with metabolic and immune pathway (**Figures 4F, G**).

### NUBPL immune infiltration analysis

**Figure 5:**
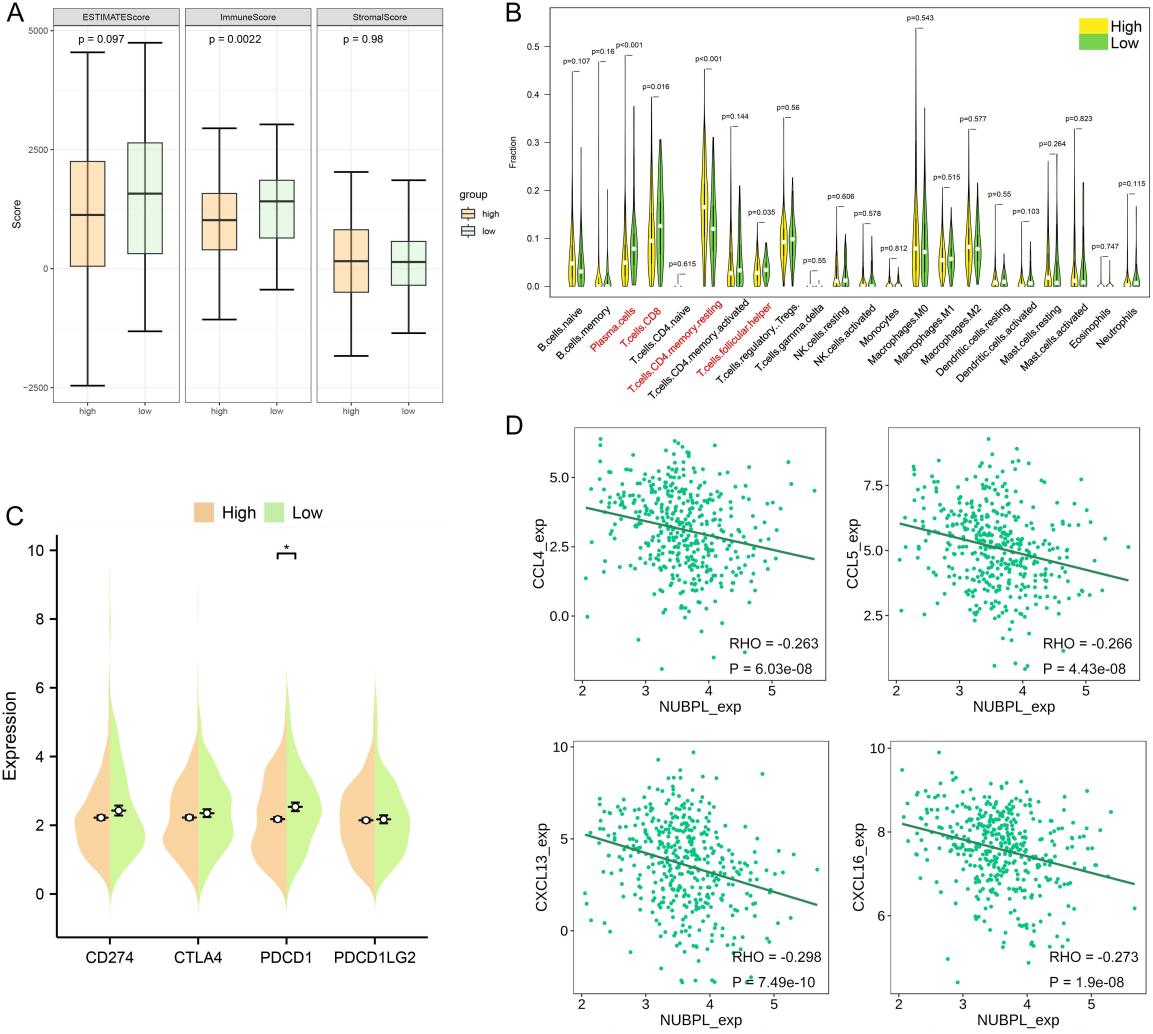
Molecular and immunological characteristics between high and low NUBPL expression cohorts. **(A)** Box plot that contrasts the NUBPL and ESTIMATE scores, as well as immune and stromal assessments. **(B)** Violin plot illustrates the disparities in immune cell infiltration between groups with high and low expression levels. **(C)** Dot plot is also presented to compare the expression of four key immune checkpoints across the same high and low groups. The statistical significance, denoted as p < 0.05, underscores the reliability of these findings. **(D)** Scatter plot reveals the association between NUBPL expressi on and the concentrations of four chemokines. *p < 0.05.

To analyze the composition of stromal and immune cells in gastric cancer samples with varying levels of NUBPL expression, the ESTIMATE algorithm was implemented. Notably, the samples with high NUBPL expression exhibited a notably higher Immune Score compared to those with low expression (**Figure 5A**). This discrepancy suggests a higher prevalence of immune cells within the high-expression group. The Immune Score clearly delineated a substantial difference between the two groups, as evidenced by the statistically significant *p*-value of 0.0022. In a bid to analyze the immune cell diversity across gastric cancer tissues with varying levels of NUBPL expression, we utilized the CIBERSORT algorithm to count the prevalence of 22 distinct tumor-infiltrating immune cell types (refer to **Supplementary Figure S3A**). The data visualized in **Figure 5B**—via a violin plot—clearly outlines the immune cell composition in groups distinguished by their NUBPL expression. A telling contrast is revealed, with the NUBPL low-expression group and the high-expression group displaying substantial discrepancies in the counts of follicular helper T cells, CD8 T cells, and plasma cells. These differences are notably significant, with CD4 T cells in resting memory phase exhibiting *p*<0.001, and plasma cells at *p*<0.05. In patients with enhanced NUBPL expression, low CD8 T cell numbers were linked to worse prognosis and accelerated tumor development.

Furthermore, we contrasted the transcript levels of four widely used immunological checkpoints between the groups expressing NUBPL at high and low levels. The two groups’ PDCD1 expression levels differed significantly, as seen in **Figure 5C**, although the high NUBPL expression cohort’s CD274, CTLA4, PDCD1, and PDCD1LG2 expression levels differed less. The findings imply that patients with elevated NUBPL expression may not benefit as much from treatment. A heatmap illustrated the correlation between NUBPL expression and 41 chemokines across pan-cancer data (**Supplementary Figure S3B**). Four molecules exhibiting statistically significant correlations were identified, indicating that the expression of CCL3 and CXCL16 was inversely correlated with NUBPL expression (**Figure 5D**). Chemokines and their receptors play an important role in the tumor microenvironment. Some studies have shown that tumor cells can secrete CXCL16 to recruit immune cells expressing CXCR6, such as NK cells and CD8^+^ T cells, to the tumor site, which can directly kill tumor cells (16). This indicates that elevated NUBPL expression may impede the function of these chemokines.

### Association analysis between NUBPL expression and immune therapy response

**Figure 6:**
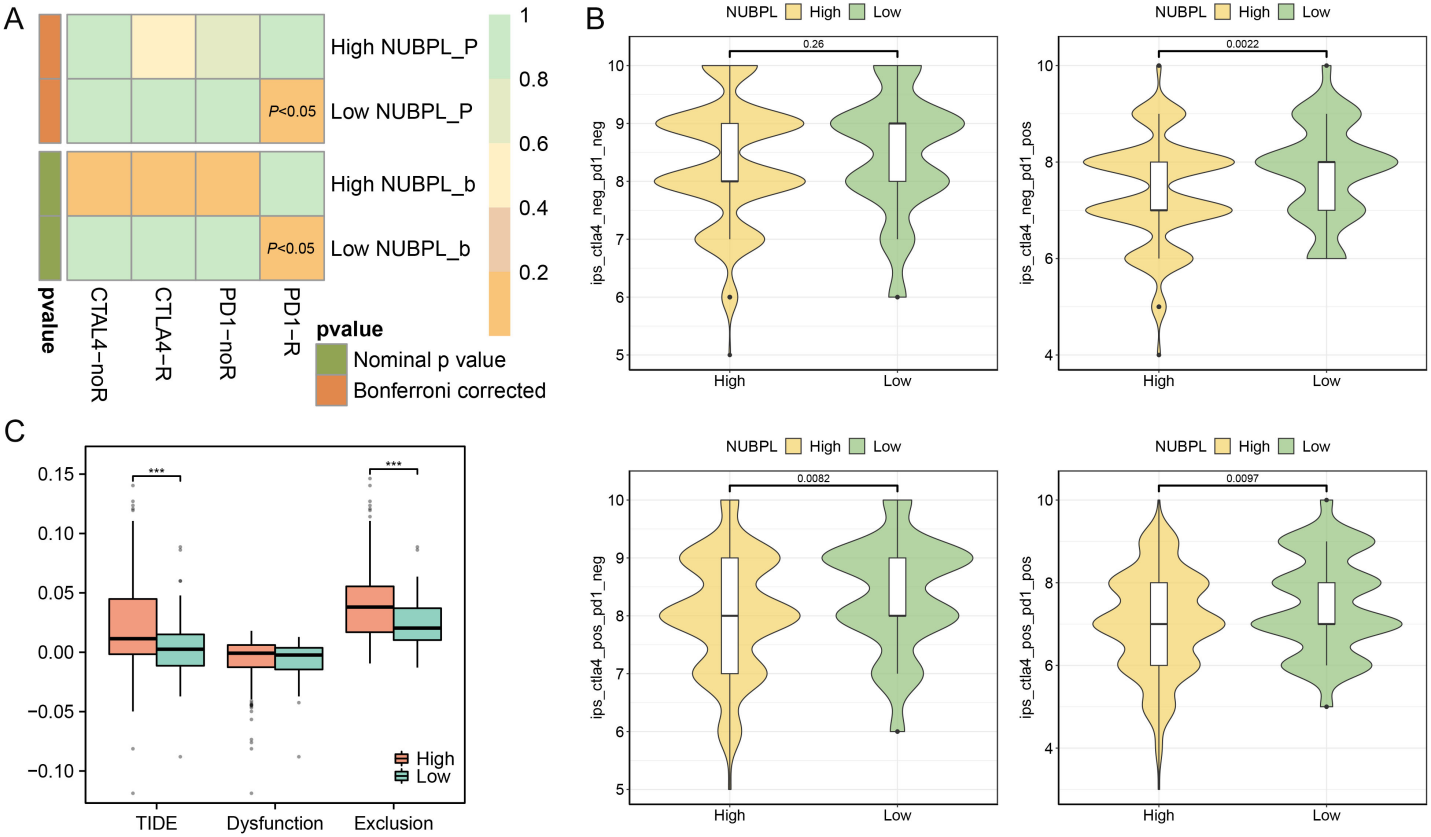
The influence of NUBPL expression on the response to immunotherapy. **(A)** SubMap analysis heatmap. **(B)** Comparison of Immunophenoscore (IPS) between high and low expression groups. **(C)** Box plot depicting the association of NUBPL levels with TIDE, T cell dysfunction, and T cell exclusion metrics; significance levels: ***p < 0.001.

We examined the relationship between NUBPL expression and immune therapy results to ascertain if NUBPL expression influences the immune therapy response in patients with gastric cancer. Patients with low NUBPL expression were more likely to respond to PD1 and CTLA-4 blocking therapies, according to the SubMap analysis (*p* < 0.05, **Figure 6A**). The group with high NUBPL expression had a significantly lower Immunophenoscore (IPS) for PD1/PDL1/PDL2 inhibitors, CTLA4 inhibitors, and the combination of CTLA4/PD1/PDL1/PDL2 inhibitors than the group with low expression (*p* < 0.05, **Figure 6B**). This implies that after receiving immune checkpoint inhibitor (ICI) medication, patients with reduced NUBPL expression can show improved therapeutic results. The exclusion of cytotoxic T lymphocytes (CTLs) and the malfunction of tumor-infiltrating CTLs are the two main processes assessed by the TIDE algorithm, which uses gene expression markers to assess tumor immune evasion mechanisms. The exclusion scores in the high NUBPL expression group were significantly higher than that in the low expression group (*p*<0.05), which also resulted in a significantly higher TIDE scores in the high NUBPL expression group (*p*<0.05, **Figure 6C**). These findings imply that the exclusion of CTLs may be the cause of the poor response to immune treatment in patients with elevated NUBPL expression. In conclusion, individuals with reduced NUBPL expression might be more vulnerable to the benefits of immunotherapy.

### Screening potential drugs targeting NUBPL

Our analysis indicates that NUBPL may be a viable target for gastric cancer treatment. We employ molecular docking technology to evaluate compounds according to their docking scores and ascertain the critical interactions of ligands within the active site of the target protein (NUBPL). Subsequently, we initiated the screening of pharmaceuticals that may effectively target NUBPL. Drug sensitivity analysis indicated that the IC50 values of multiple drugs, such as QS11, Imatinib, AS601245, Sorafenib, and Tipifarnib, were markedly reduced in the high NUBPL expression cohort relative to the low expression cohort (*p* < 0.05, **Supplementary Figure S4**). This signifies a positive correlation between NUBPL expression levels and sensitivity to these pharmaceuticals.

**Figure 7:**
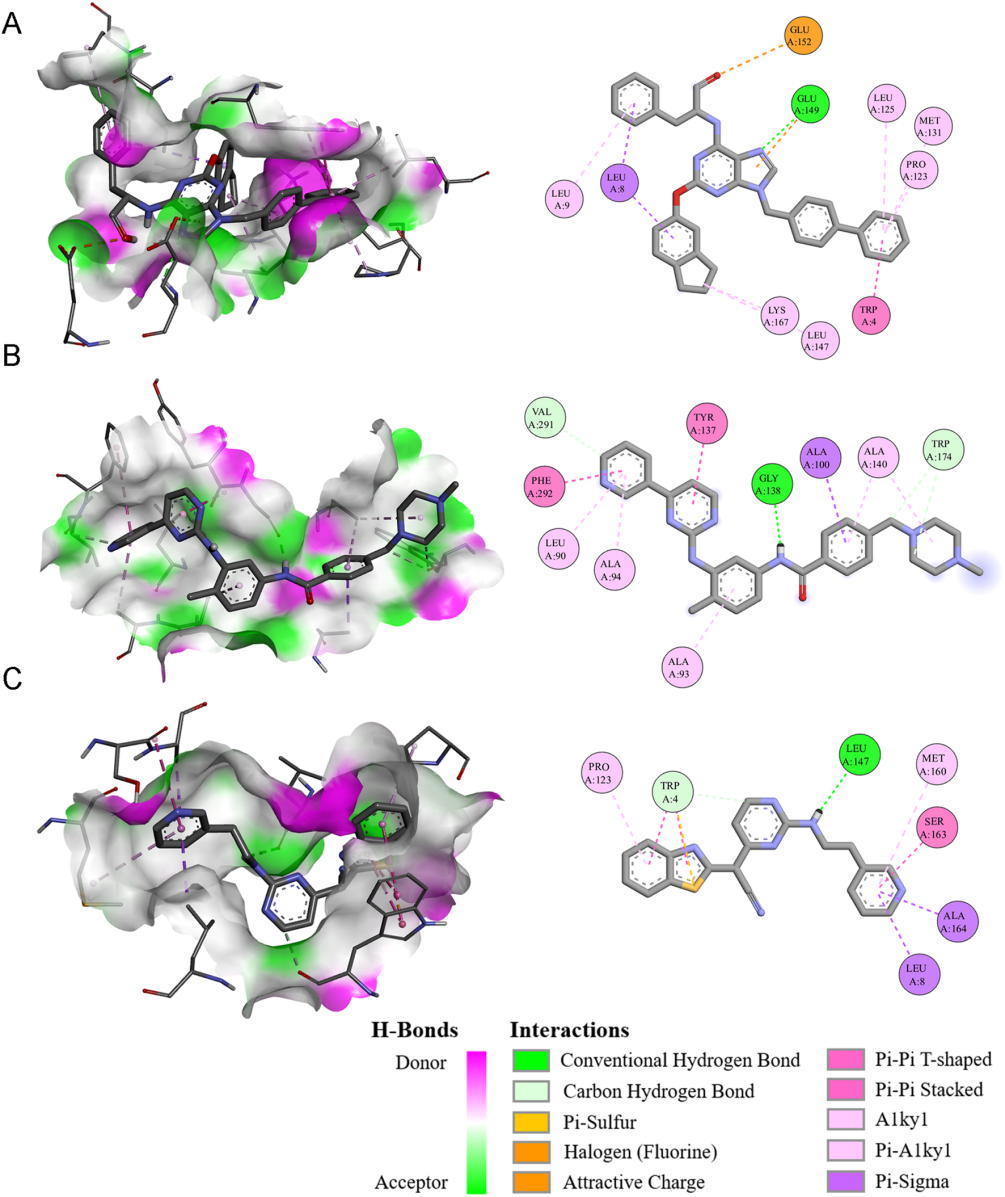
The three-dimensional and two-dimensional interaction diagrams of the NUBPL protein with QS11 **(A)**, Imatinib **(B)**, and AS601245 **(C)**.

We conducted a detailed examination of the binding affinities between these compounds and the NUBPL protein through molecular docking analysis. The findings indicated that QS11, Imatinib, and AS601245 exhibited binding free energies below -8.0 kcal/mol (**Table 1**), implying that these three compounds interact more stably with the NUBPL protein. QS11 is a purine derivative that regulates the Wnt/β-catenin signaling pathway by influencing protein transport. It can inhibit the migration of breast cancer cells with ARFGAP overexpression (17). Imatinib is a potent tyrosine kinase inhibitor that specifically targets the BCR-ABL, KIT, and PDGFR kinases. It is used for the treatment of chronic myeloid leukemia (CML), gastrointestinal stromal tumors (GIST), and other types of cancers (18). AS601245 is a selective JNK inhibitor, which has the effects of inhibiting cell adhesion and migration in colon cancer cells (19). **Figure 7** illustrates the identified hydrogen bonds and hydrophobic interactions. Furthermore, we investigated the particular interactions among these three drugs and the NUBPL protein. Each compound establishes hydrogen bonds with NUBPL: GLU149 interacts with QS11 (**Figure 7A**), VAL291, GLY138, and TRP174 interact with Imatinib (**Figure 7B**), while TRP4 and LEU147 interact with AS601245 (**Figure 7C**). The results suggest that QS11, Imatinib, and AS601245 may inhibit NUBPL protein activity, positioning them as promising candidates for further research.

**Table 1:** Candidates bind to the fragments of NUBPL protein for drug repositioning.

Drugs	Binding affinity (Kcal/mol)	Groups	Chemical Formula	Indication
QS11	-9.8	Investigational	C_36_H_33_N_5_O_2_	QS11, an ARFGAP1 inhibitor, enhances Wnt/β-catenin signaling by affecting protein trafficking and inhibits migration of ARFGAP-overexpressing breast cancer cells.
Imatinib	-8.5	Approved	C_29_H_31_N_7_O	Lmatinib is a tyrosine kinase inhibitor used to treat a number of leukemias, myelodysplastic disease, systemic mastocytosis, hypereosinophilic syndrome, dermatofibrosarcoma protuberans, and gastrointestinal stromal tumors.
AS601245	-8.2	Investigational	C_20_H_16_N_6_S	AS601245 is an orally active, selective, ATP-competitive JNK inhibitor. It has neuroprotective properties and is an anti-inflammatory agent that has been associated with nerve damage.
Sorafenib	-7.9	Approved, Investigational	C_21_H_16_ClF_3_N_4_O_3_	Sorafenib is a kinase inhibitor used to treat unresectable liver carcinoma, advanced renal carcinoma, and differentiated thyroid carcinoma.
Tipifarnib	-7.4	Investigational	C_27_H_22_Cl_2_N_4_O	Investigated for use/treatment in colorectal cancer, leukemia (myeloid), pancreatic cancer, and solid tumors.
Lapatinib	-7.4	Approved, Investigational	C_29_H_26_ClFN_4_O_4_S	Lapatinib is an antineoplastic agent and tyrosine kinase inhibitor used for the treatment of advanced or metastatic HER-positive breast cancer in patients who received prior chemotherapeutic treatments.
Doxorubicin	-7.1	Approved, Investigational	C_27_H_29_ NO_11_	Doxorubicin is a medication used to treat various cancers, including AIDS-associated Kaposi’s Sarcoma and metastatic cancers.
Erlotinib	-6.6	Approved	C_22_H_23_N_3_O_4_	It is an EGFR tyrosine kinase inhibitor used to treat certain small cell lung cancers or advanced metastatic pancreatic cancers.
Shikonin	-6.5	Investigational	C_16_H_16_O_5_	Shikonin is used in the treatment of acute jaundiced or non-jaundiced hepatitis, chronic hepatitis and flat warts. It is also effective for liver cirrhosis (ascites) and common warts.
AZD6482	-6.4	Investigational	C_22_H_24_N_4_O_4_	It is a selective inhibitor of PI3Kβ (phosphatidylinositol 3-kinase beta), which has shown potential therapeutic effects in clinical studies for a variety of diseases.
Gemcitabine	-6.0	Approved	C_9_H_11_F_2_N_3_O_4_	Gemcitabine is a nucleoside metabolic inhibitor used as adjunct therapy in the treatment of certain types of ovarian cancer, non-small cell lung carcinoma, metastatic breast cancer, and as a single agent for pancreatic cancer.
Embelin	-5.5	Investigational	C_17_H_26_O_4_	Isolated from Lysimachia punctata and Embelia ribes, it exhibits antimicrobial, antineoplastic and inhibitory activity towards hepatitis C protease. It has a role as a hepatitis C protease inhibitor, an antimicrobial agent, an antineoplastic agent and a plant metabolite.

### The effect of NUBPL on the invasion and migration of gastric cancer cells

**Figure 8:**
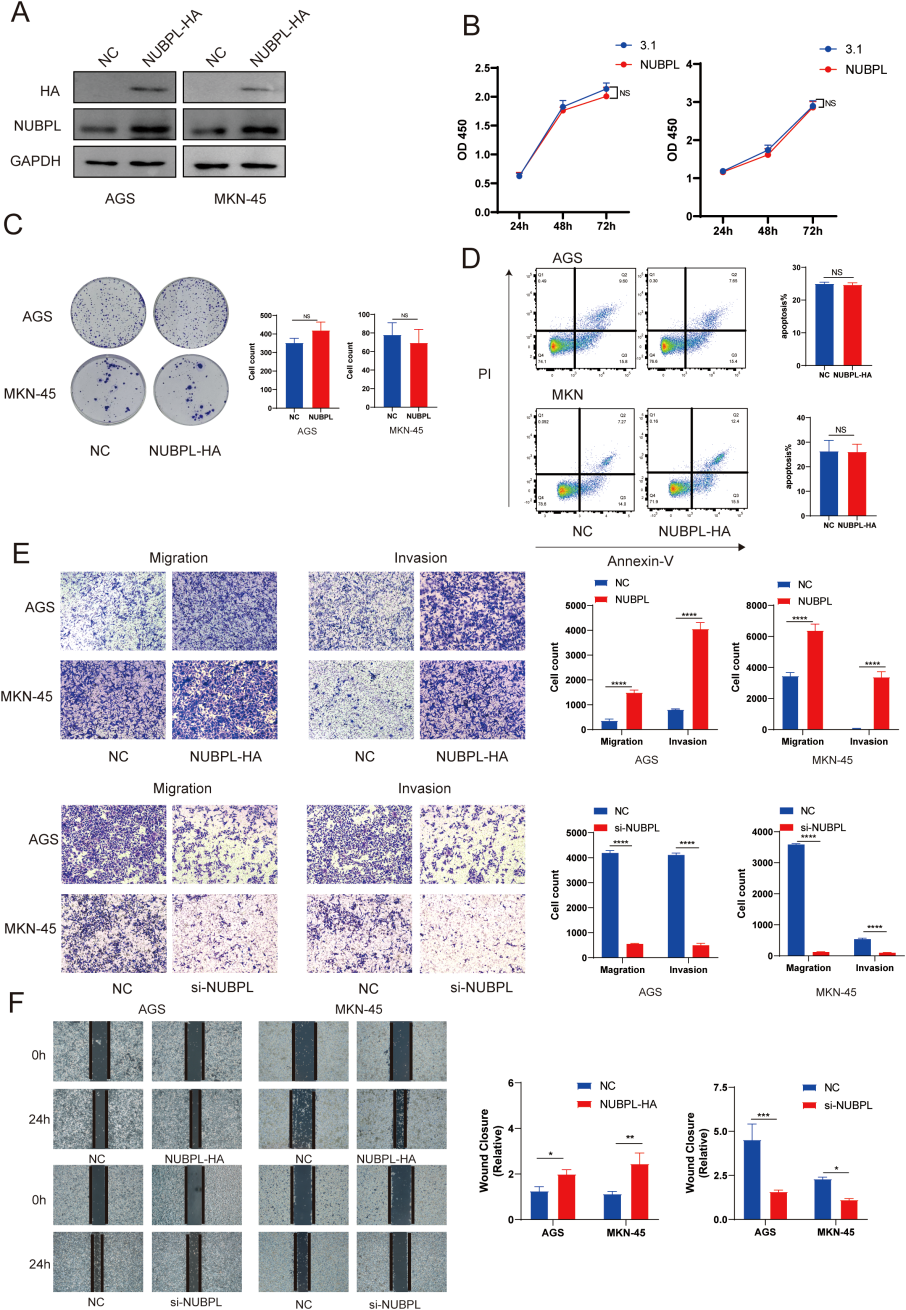
The impact of NUBPL on gastric cancer cell phenotype. **(A)** Western blotting assessment of NUBPL protein levels in AGS and MKN cell lines. **(B)** CCK8 assay for cellular proliferation. **(C)** Colony formation assay. **(D)** Annexin-V/PI assay for cellular apoptosis. **(E)** Transwell assay for cellular invasion and migration. **(F)** Cell scratch assay. ns, not significant; *p < 0.05; **p < 0.01; ***p < 0.001.

To detect the effect of NUBPL on the malignant phenotype of gastric cancer, we overexpressed NUBPL in GC cell lines AGS and MKN-45, and confirmed the overexpression efficiency through Western blotting (**Figure 8A**). Our results showed that NUBPL overexpression did not affect cell proliferation (**Figure 8B**), and colony formation assays further confirmed that NUBPL had no impact on cell growth (**Figure 8C**). Additionally, we found that NUBPL did not influence apoptosis in gastric cancer cells (**Figure 8D**). Transwell assays have shown that NUBPL notably drives the aggression and dispersal of stomach cancer cells. In contrast, knocking down NUBPL with siRNA significantly inhibits the invasion and migration of gastric cancer cells compared to the control group, as depicted in **Figure 8E**. Furthermore, scratch wound healing experiments indicate that NUBPL’s elevated levels accelerate the movement of these cells, knocking down NUBPL inhibits the movement of cells. (**Figure 8F**).

### The effect of NUBPL on disulfidptosis in gastric cancer cells

As a result of our analysis, we delved into the expression of SLC7A11 in various gastric cancer cell lines, and we found that SLC7A11 protein levels surged across the board in these lines, with the sole exception being the HGC cells (**Supplementary Figure S5A**). Following this discovery, we conducted experiments with AGS and MKN-45 cells grown in a glucose-free environment. We tested the efficacy of cell death pathway inhibitors, all of which were unable to thwart cell demise, whereas the disulfide death inhibitor TCEP was able to reverse cell death. This indicates that AGS and MKN-45 exhibit a novel form of cell death triggered by glucose deprivation (**Supplementary Figure S5B**). A key indicator of disulfidptosis is the cross-linking of disulfide bonds in scaffold proteins. In conditions of glucose deprivation, specifically after approximately 24 hours, the migration of the actin cytoskeletal protein ACTB in AGS and MKN-45 cell lines is markedly diminished. The effect is suppressed in the absence of glucose and in the presence of TCEP intervention (**Supplementary Figure S5C**). Liu’s research revealed that abnormal disulfide bonds in the actin cytoskeleton protein of SLC7A11high cells, triggered by glucose deprivation, could lead to later F-actin contraction and separation from the plasma membrane. Glucose deprivation caused substantial alterations in cell morphology, marked by cell and actin contraction, which were reversible by TCEP (**Supplementary Figure S5D**).

**Figure 9:**
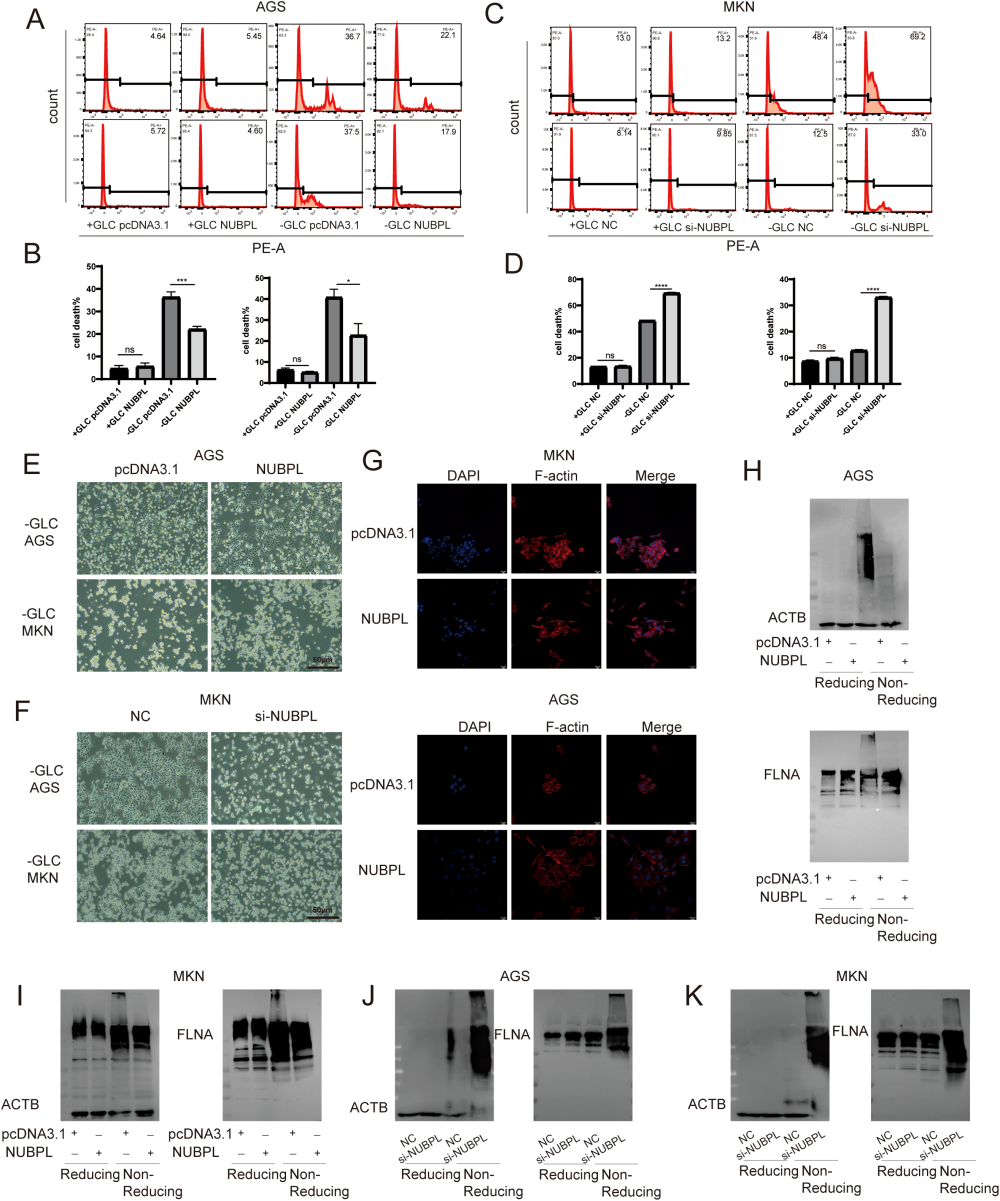
The impact of NUBPL on disulfide-induced mortality in gastric cancer cells. **(A-D)** The influence of NUBPL on disulfidptosis in gastric cancer cells under conditions of glucose supplementation and deprivation. **(E, F)** Observation of cell morphology via microscopy. **(G)** Immunofluorescence assessment of F-actin expression. **(H-K)** Detection of disulfide bonds in non-reducing SDS-PAGE. ns, not significant; *p < 0.05; ***p < 0.001; ****p < 0.0001.

To evaluate the impact of NUBPL on gastric cancer cells, we employed PI staining to quantify cell mortality and discovered that NUBPL overexpression markedly diminished cell death during glucose deprivation (**Figures 9A, B**). In contrast, depletion of NUBPL by siRNA resulted in a higher cell death rate, compared to those in the control cells (**Figures 9C, D**, **Supplementary Figure S6**). Under observation with an inverted microscope, under glucose-free conditions, cells in the pcDNA3.1 group exhibited characteristics of dull appearance and membrane fragmentation. In contrast, cells in the NUBPL group were able to re-adhere, with intact cell membranes. Conversely, cells with NUBPL knockdown showed the opposite trend (**Figures 9E, F**). Simultaneously, the application of ghost pen cyclic peptide staining revealed that the overexpression of the NUBPL group markedly enhanced cell morphology under glucose-free conditions (**Figure 9G**). Subsequent analysis verified that the disulfide bond tailing of actin was markedly diminished in the NUBPL overexpression group relative to the NC group during non-reducing electrophoresis however, the disulfide bond tailing of actin was significantly increased in the NUBPL knockdown group compared to the NC group (**Figures 9H-K**).

### NUBPL activates the pentose phosphate pathway to inhibit disulfidptosis

**Figure 10:**
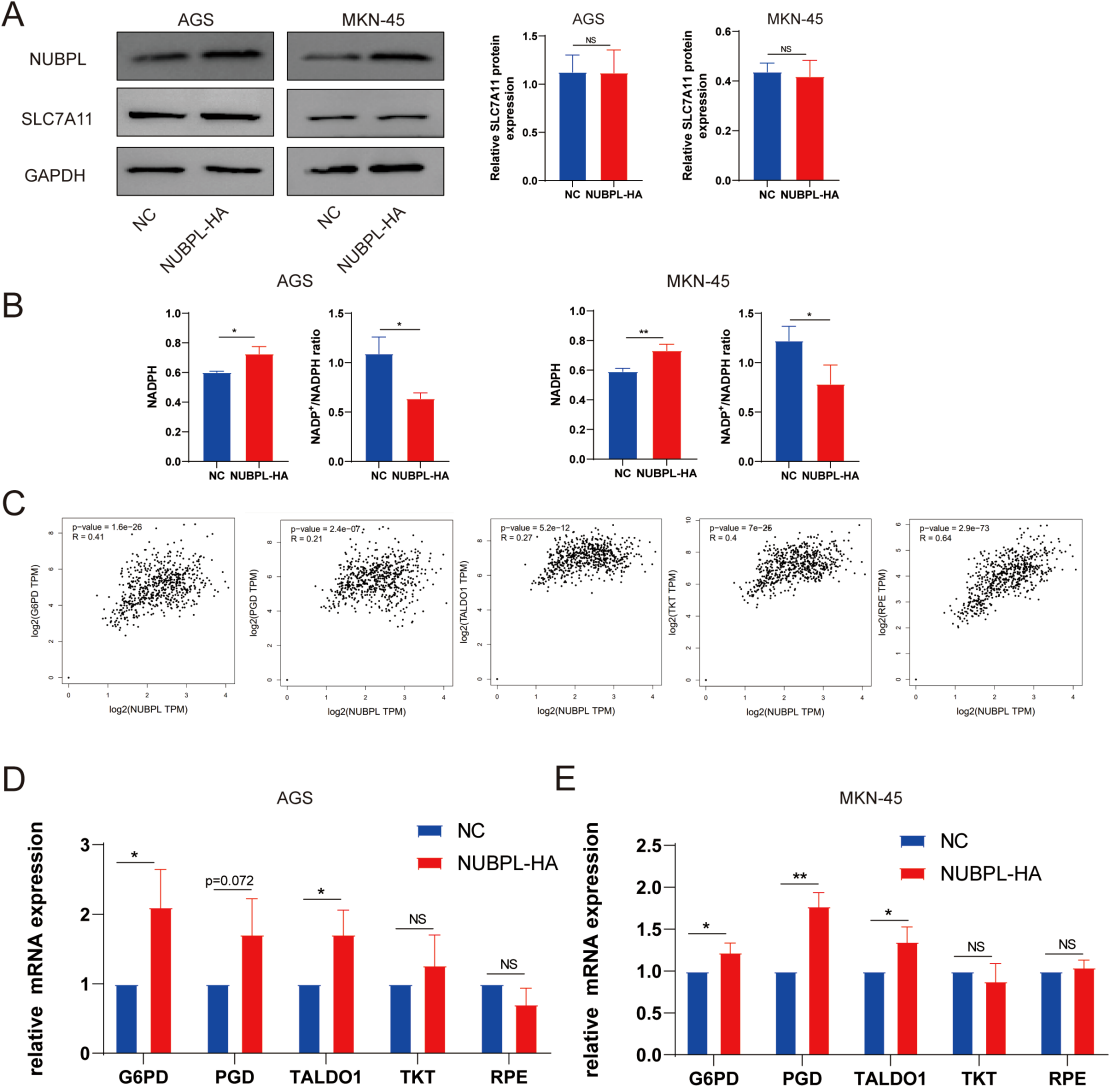
The regulatory mechanism of NUBPL in disulfide-induced apoptosis in gastric cancer cells. **(A)** Western blot analysis of SLC7A11 expression. **(B)** Measurement of the NADP^+^/NADPH ratio. **(C)** Correlation of NUBPL with pivotal enzymes in the PPP pathway (G6PD, PGD, RPE, TALDO1, TKT). **(D)** Quantitative PCR analysis of the impact of NUBPL on the expression of enzymes in the pentose phosphate pathway. ns, not significant; *p < 0.05; **p < 0.01.

We examined the effect of NUBPL on SLC7A11 expression and found no significant effect (**Figure 10A**), which is inconsistent with the hypothesis that increased SLC7A11 expression promotes disulfidptosis. The correlation between disulfide mortality and NADPH depletion was brought to light by Liu et al. Cysteine absorption is enhanced in cells expressing large levels of SLC7A11, and NADPH is necessary for the conversion of cystine to cysteine. Intracellular NADPH is mostly produced by the pentose phosphate pathway (PPP). In a glucose-free environment, SLC7A11 cells experience an imbalance between NADPH supply and consumption, leading to an aberrant buildup of cystine, an increase in disulfide bond stress, and ultimately, cell death caused by disulfide. It appears that NUBPL may mitigate disulfide-induced cell death by reducing NADP^+^/NADPH levels, since the overexpressed NUBPL group exhibited noticeably lower amounts compared to the NC group (**Figure 10B**). To delve deeper into the matter, we examined the association between NUBPL and genes associated to NADPH using Spearman correlation analysis. **Figure 10C** shows that there was a positive correlation between NUBPL expression and G6PD, PGD, RPE, TALDO1, and TKT. Overexpression of NUBPL was found to activate PPP essential enzymes by qPCR analysis (**Figure 10D**), suggesting that NUBPL might modulate the PPP pathway to prevent disulfidptosis.

## Discussion

Gastric cancer is a highly prevalent malignant tumor. The number of newly diagnosed cases worldwide exceeds one million every year, mainly distributed in East Asia and South America (20–22). The survival probability at 5 years for patients with advanced gastric carcinoma is under 30%, despite recent improvements in endoscopy, imaging, surgery, and anticancer pharmacotherapy that have marginally improved the overall prognosis (23, 24). Copy number modifications resulted in the amplification of genes associated with the tyrosine kinase receptor pathway, including FGFR2, HER2, EGFR, MET, and WNT (25, 26). Consequently, the identification of early molecular markers for gastric cancer may facilitate the development of molecular targeted therapies, prognostic tools, and early detection strategies (27, 28). NUBPL was identified as a potential biomarker for prognosis and immunotherapy in gastric cancer patients through bioinformatics analysis in the study. NUBPL is a component of the respiratory chain dehydrogenase complex, which is associated with mitochondrial metabolism, and is assembled within the inner mitochondrial membrane (29, 30). The majority of NUBPL research has concentrated on neurological disorders, with only a small number of studies investigating malignancies (31). Previous research has demonstrated that colorectal cancer cells express NUBPL at a high level, and that elevated NUBPL levels can promote metastasis and epithelial-mesenchymal transition (EMT) (32). The function of NUBPL in gastric carcinoma has been the subject of limited prior research. In order to address this lacuna, we conducted an analysis of data from numerous databases to determine the potential impact of NUBPL on gastric cancer.

The immune microenvironment in gastric cancer is pivotal in tumor proliferation, metastasis, and treatment efficacy. Diverse cell types in the tumor microenvironment, including tumor-associated macrophages, regulatory T cells, and dendritic cells, engage in intricate interactions that either facilitate or impede tumor progression (33). Consequently, a more profound comprehension of the interactions among these cells may uncover novel therapeutic targets and inspire innovative approaches for immunotherapy in gastric cancer (34, 35). Alterations in the immune microenvironment are critical determinants of tumor progression, as tumor cells exploit immune cell infiltration to circumvent immune surveillance (36, 37). There is a study showing that in bladder cancer, high expression of NUBPL leads to a poor response to immune checkpoint inhibitors (ICI) (38). We found that the aberrant expression of NUBPL markedly influences the metabolic pathways and immune microenvironment of gastric cancer, especially its impact on immune cell infiltration. Our analysis indicates that NUBPL may contribute to immune evasion by affecting immune-related pathways.

This study also evaluated various candidate drugs aimed at NUBPL, including QS11, Imatinib, and AS601245. These medications may produce therapeutic effects by inhibiting the function of the NUBPL protein. Subsequent research should validate the efficacy of these pharmaceuticals in the treatment of gastric cancer, specifically by assessing their anti-tumor properties in preclinical models and investigating the potential for synergistic use with other immunotherapeutic agents (38).

Metabolic reprogramming is a critical characteristic of cancer, frequently leading to the development of a strong dependence on specific nutrients or metabolic pathways by cancer cells. In the era of precision oncology, it has become increasingly common to target cancer metabolism in order to selectively eliminate cancer cells (39). In the treatment of cancer metabolism, regulatory cell death (RCD) is essential (40). Disulfidptosis, a novel form of metabolically regulated cell death, was recently identified by Xiaoguang Liu and colleagues (2). Previous research has shown that the SLC7A11 protein has a substantial impact on disulfide death. Specifically, cells with a high level of SLC7A11 expression are more susceptible to disulfide death (41). We identified numerous gastric cancer cell lines and observed a substantial increase in slc7a11. Simultaneously, we administered a sugar-free treatment and found that its mortality could only be suppressed by TCEP, thereby excluding other inhibitors of death pathways. The results indicated that disulfide death was the mode of death in gastric cancer in the absence of sugar. BAY876 has the ability to replicate sugar-free conditions and inhibit glucose transporters (42). Leveraging this property, we can combine oxaliplatin and other chemotherapeutic agents with BAY876 in the treatment of gastric cancer, potentially increasing efficacy. NUBPL is an assembly factor for human mitochondrial complex I, the largest component of the mitochondrial respiratory chain. The role of NUBPL in disulfide-induced mortality remains unknown. Our findings identified NUBPL as a pro-oncogene that inhibits apoptosis in a sugar-deprived environment, potentially explaining the poor prognosis in patients with elevated NUBPL expression. The pentose phosphate pathway (PPP) is critical for disulfide-induced cell death. During glucose deprivation, renal cancer cells with increased SLC7A11 gene expression depleted NADPH within an hour, resulting in an increase in NADP^+^/NADPH levels (2). Overexpression of NUBPL had no effect on SLC7A11 expression levels. NADP^+^/NADPH levels were lower than those in the control group, and our findings indicate that NUBPL influenced the expression of enzymes associated with the PPP pathway, suggesting that NUBPL may inhibit disulfide-induced cell death via the PPP pathway.

However, our study has certain limitations. For example, the sample size is relatively small and the sources of the databases are limited. The accuracy of the prognostic model derived from public databases requires further experimental validation, such as constructing immunohistochemical chips for gastric cancer to further analyze the prognostic value of NUBPL in gastric cancer. Secondly, the mechanism by which NUBPL inhibits disulfide death needs to be further explored. Finally, since there is limited research on the NUBPL gene, we can also analyze its role from a pan-cancer perspective to provide a theoretical basis for NUBPL as a potential tumor marker.

## Conclusion

We have confirmed that NUBPL is upregulated in gastric cancer and is associated with poor prognosis, while also influencing immune infiltration and treatment of gastric cancer cells. *In vitro*, NUBPL affects the invasion and migration of gastric cancer cells, but not proliferation and apoptosis. We also found that NUBPL inhibits disulfidptosis in gastric cancer cells. Additionally, we discovered that NUBPL is positively correlated with key enzymes of the pentose phosphate pathway (PPP) and that NUBPL can influence the PPP. Therefore, NUBPL can serve as a key biomarker for gastric cancer, providing a new potential target for targeted therapy and immunotherapy.

## Data Availability

The datasets presented in this study can be found in online repositories. The names of the repository/repositories and accession number(s) can be found in the article/**Supplementary Material**.

## Supplementary material

Supplementary Figure 1 NCKAP1 and NUBPL Expression in Gastric Cancer vs. Healthy Tissue.

Supplementary Figure 2 The volcano graph illustrates the varied expression of genes in the NUBPL high and low expression subsets.

Supplementary Figure 3 Immune Cells and Chemokine Analysis in Gastric Cancer. **(A)** Percentage of Immune Cells Identified in Gastric Cancer Tissue Samples. **(B)** Heatmap of the correlation between NUBPL expression and chemokines across pan-cancer data.

Supplementary Figure 4 Box plot illustrating NUBPL drug sensitivity.

Supplementary Figure 5 The impact of gastric cancer cells under non-sugar-rich conditions. **(A)** SLC7A11 Expression in Gastric Cancer Cell Cultures. **(B)** Effect of cell death inhibitors on gastric cancer cells in sugar-free condition. **(C)** Detection of disulfide bonds in non-reducing SDS-PAGE. **(D)** Immunofluorescence detection of F-actin expression.

Supplementary Figure 6 Western blotting analysis of NUBPL of AGS and MKN cells expressing depleted of NUBPL by siRNA.
