# Supplementary figures and images for: Multidisciplinary analysis of the prognosis and biological function of NUBPL in gastric cancer
Source: Front Immunol. 2025 Jun 5;16:1603898. doi: 10.3389/fimmu.2025.1603898 (PMC12176871; doi:10.3389/fimmu.2025.1603898)

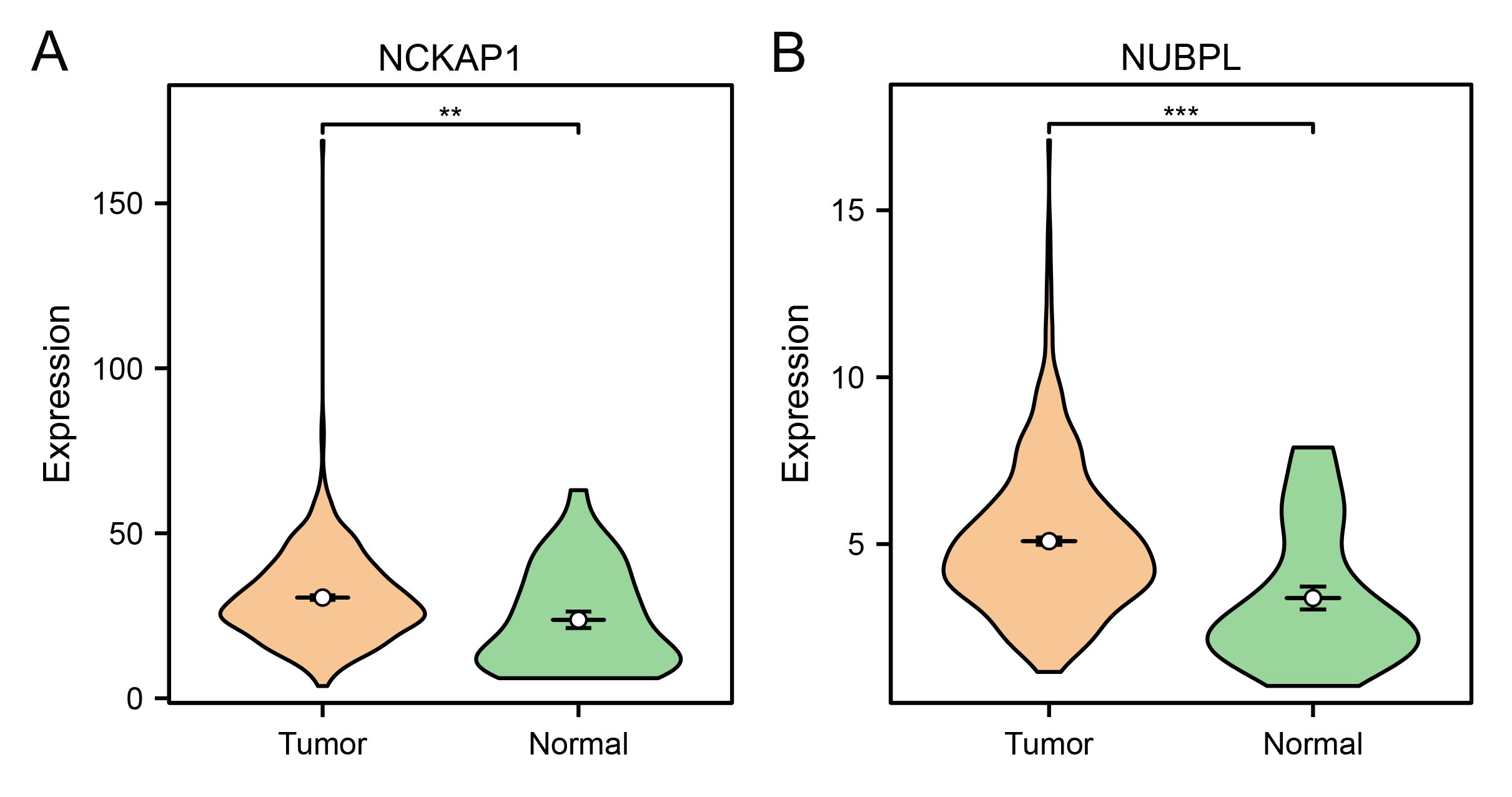

Supplement: Supplementary Figure 1 — NCKAP1 and NUBPL Expression in Gastric Cancer vs. Healthy Tissue. [file Image1.tif]

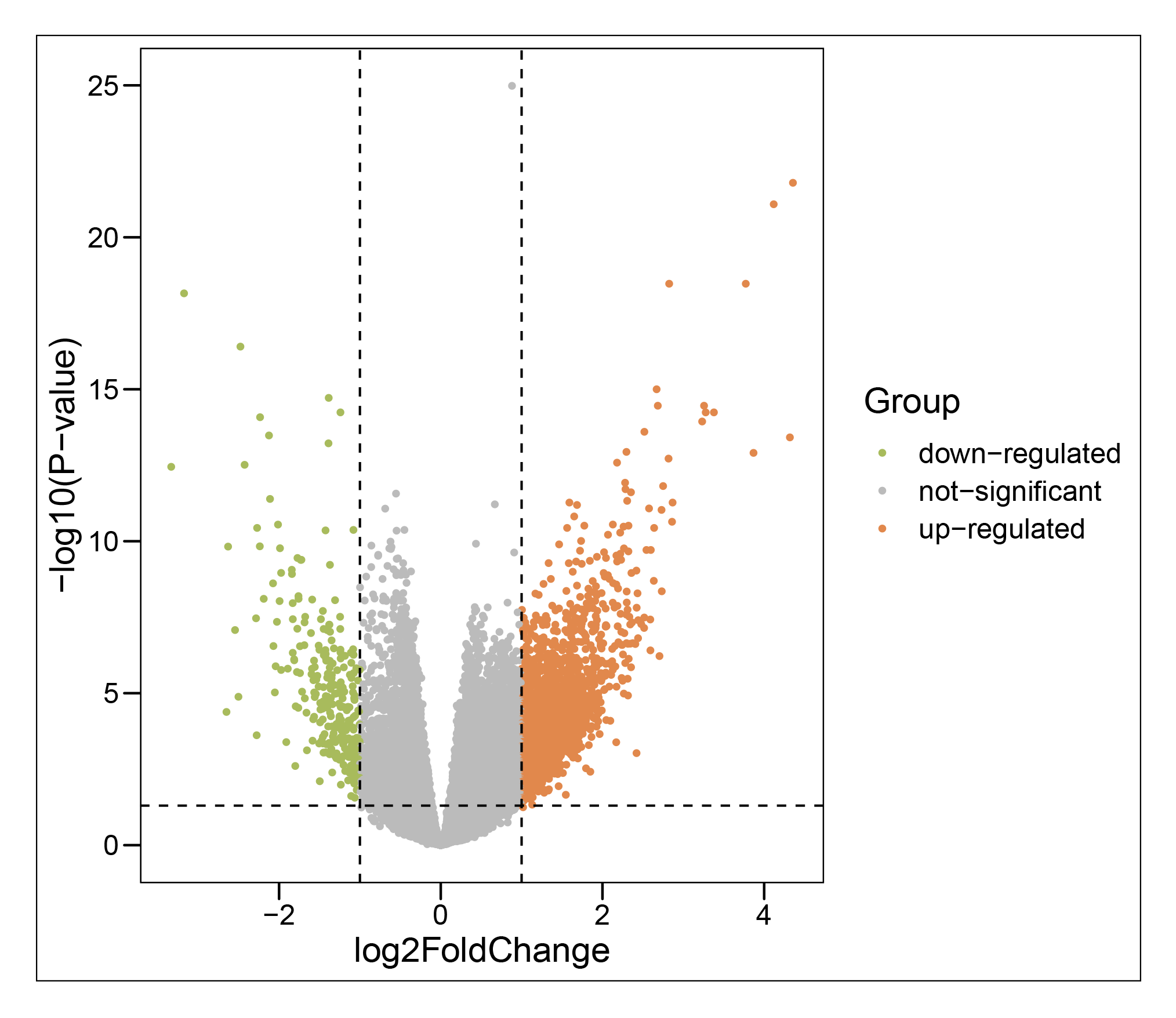

Supplement: Supplementary Figure 2 — The volcano graph illustrates the varied expression of genes in the NUBPL high and low expression subsets. [file Image2.tif]

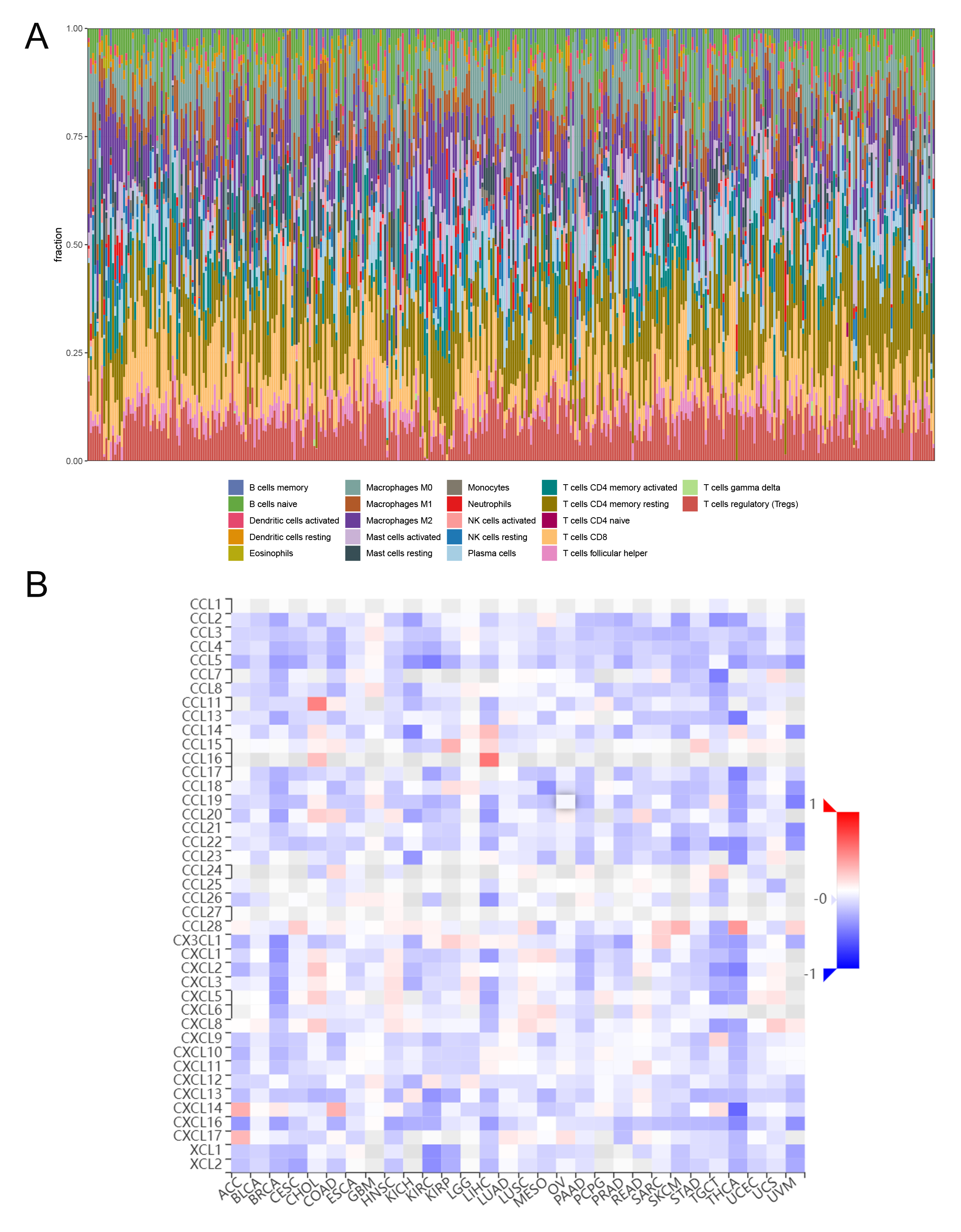

Supplement: Supplementary Figure 3 — Immune Cells and Chemokine Analysis in Gastric Cancer. (A) Percentage of Immune Cells Identified in Gastric Cancer Tissue Samples. (B) Heatmap of the correlation between NUBPL expression and chemokines across pan-cancer data. [file Image3.tif]

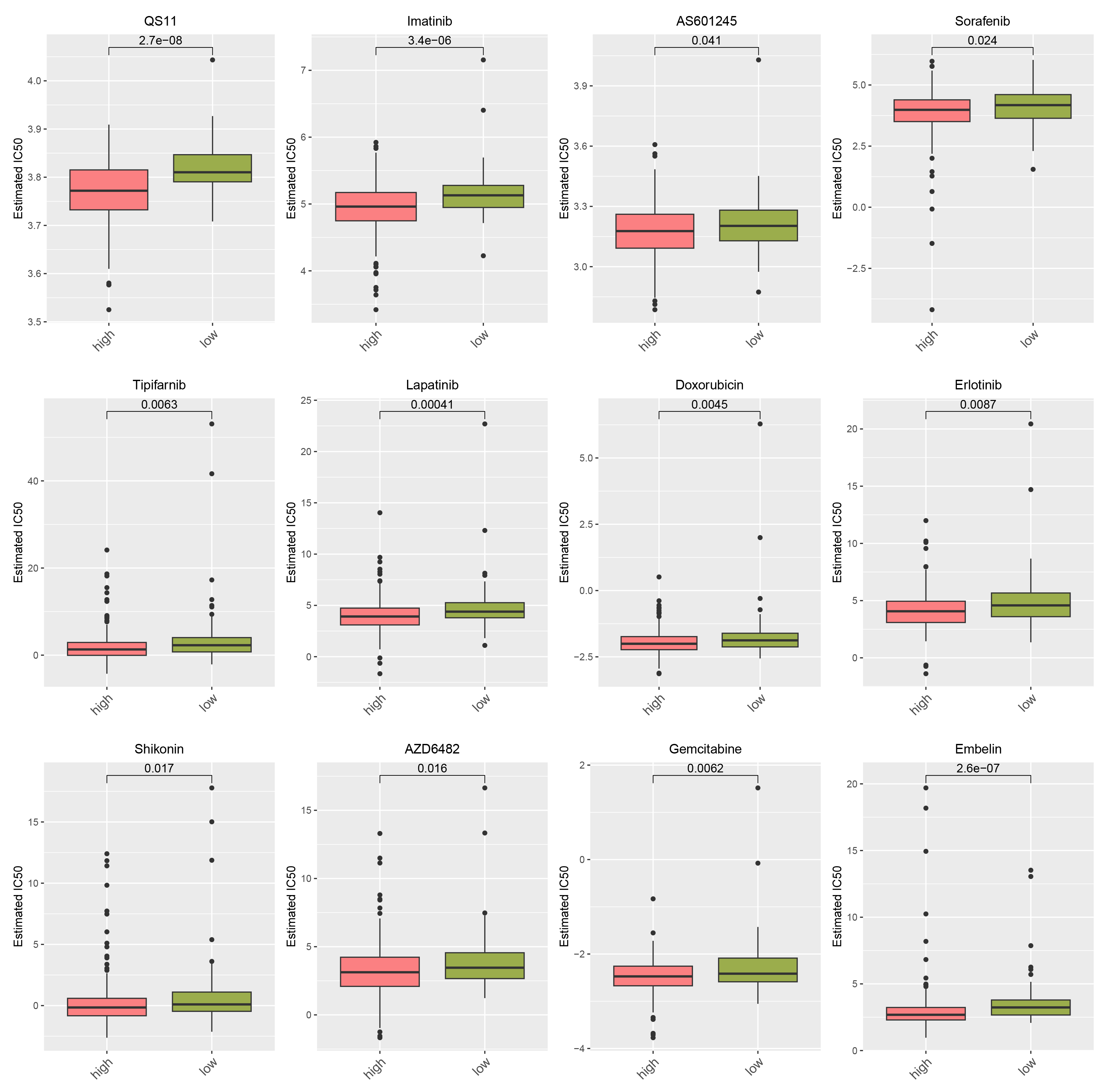

Supplement: Supplementary Figure 4 — Box plot illustrating NUBPL drug sensitivity. [file Image4.tif]

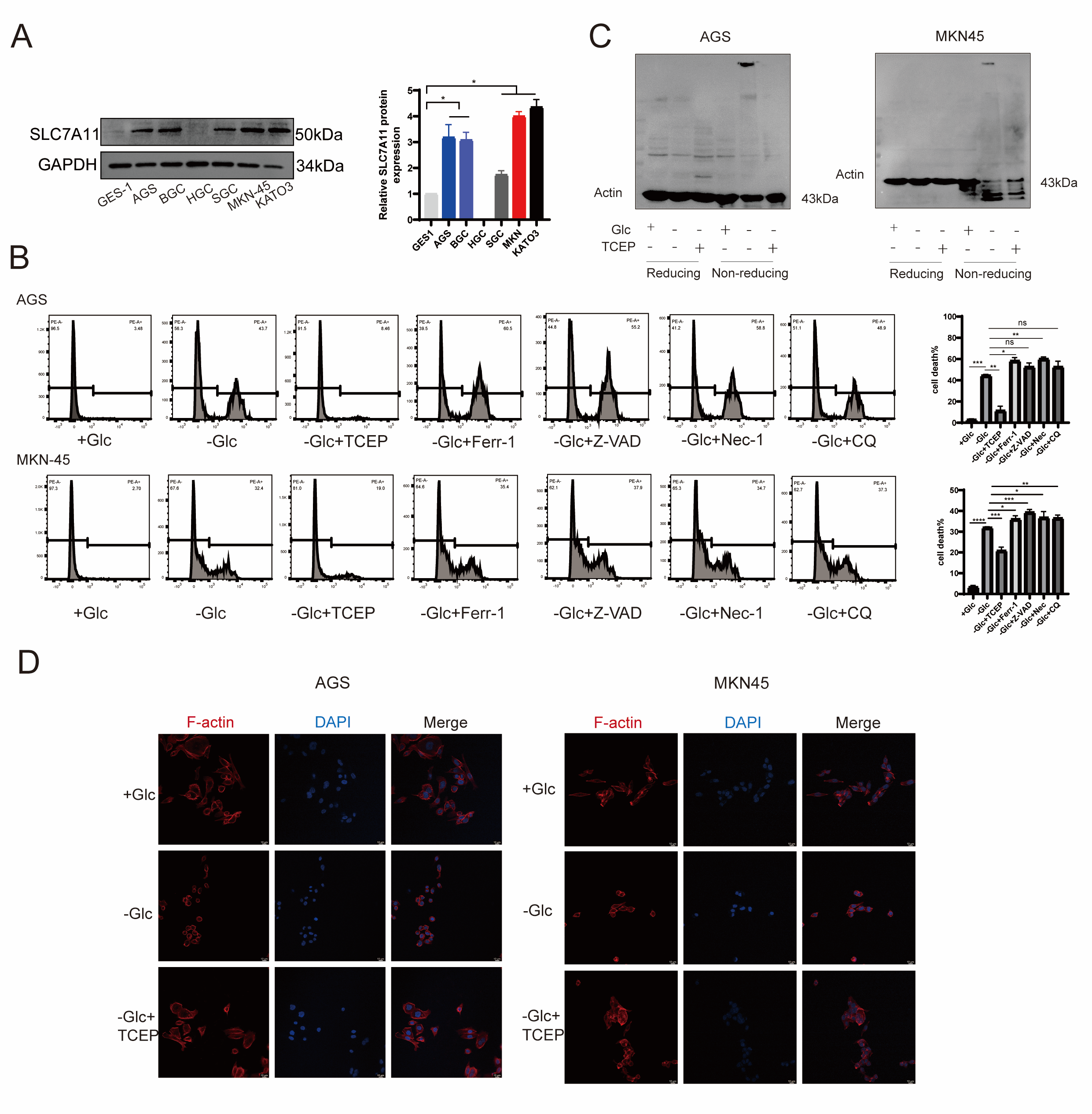

Supplement: Supplementary Figure 5 — The impact of gastric cancer cells under non-sugar-rich conditions. (A) SLC7A11 Expression in Gastric Cancer Cell Cultures. (B) Effect of cell death inhibitors on gastric cancer cells in sugar-free condition. (C) Detection of disulfide bonds in non-reducing SDS-PAGE. (D) Immunofluorescence detection of F-actin expression. [file Image5.tif]

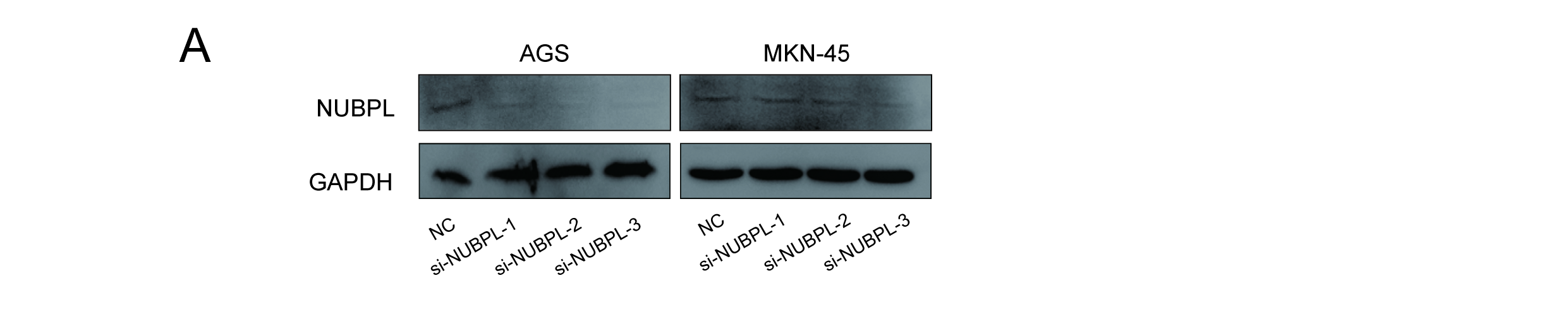

Supplement: Supplementary Figure 6 — Western blotting analysis of NUBPL of AGS and MKN cells expressing depleted of NUBPL by siRNA. [file Image6.tif]
